# Waste Quartz Sand as a Sustainable Modifier for Bituminous Binders: An Experimental Performance Assessment

**DOI:** 10.3390/polym18192424

**Published:** 2026-10-05

**Authors:** İmam Çalış, Murat Vergi Taciroğlu

**Affiliations:** 1 Institute of Science, Department of Civil Engineering, Mersin University, 33343 Mersin, Türkiye; immcls02@gmail.com; 2 Faculty of Engineering, Department of Civil Engineering, Mersin University, 33343 Mersin, Türkiye

**Keywords:** waste quartz sand, bitumen modification, rheological properties, aging behavior, adhesion, sustainable road materials

## Abstract

Waste quartz sand (WQS), a silica-rich by-product of the glass manufacturing industry, is commonly disposed of, creating environmental and resource-management concerns. This study addresses the limited knowledge regarding the use of WQS as a mineral-based modifier for bituminous binders. Fine WQS (<0.075 mm) was incorporated into B50/70 penetration-grade bitumen at 0%, 1%, 2%, 3%, and 4% by weight. The morphology and chemical composition of WQS were characterized, and the modified binders were evaluated for conventional physical properties, storage stability, temperature-dependent rheological response, short- and long-term aging behavior, low-temperature performance, and aggregate–binder adhesion. WQS incorporation decreased penetration and increased the softening point while maintaining satisfactory storage stability. A 2% WQS content provided the most favorable high-temperature rheological performance and rutting-related response. Low-temperature performance was largely maintained, while adhesion performance remained satisfactory at 1–3% WQS but decreased at 4% WQS. Overall, 2% WQS exhibited the most favorable balance among the evaluated binder properties. The screening-level environmental and economic assessment indicated potential reductions in virgin bitumen consumption, greenhouse gas emissions, and binder cost. These findings demonstrate the potential of finely processed WQS as a waste-derived mineral modifier for bituminous binders and provide an alternative pathway for the valorization of glass-industry waste in pavement materials.

## 1. Introduction

Bitumen is one of the key components governing the durability and service life of flexible pavements. However, due to its temperature-sensitive viscoelastic behavior, conventional bitumen may not consistently deliver adequate performance under varying environmental conditions and traffic loading [1,2,3,4]. High temperatures can promote permanent deformation, whereas aging, moisture exposure, and low-temperature conditions may contribute to different forms of pavement deterioration [4,5,6,7]. To address these limitations, various modification approaches involving polymers, rubbers, nanomaterials, and waste-derived materials have been investigated to improve the physical, mechanical, and rheological properties of bituminous binders [3,8,9,10,11]. Although polymeric and other conventional modifiers can improve resistance to rutting, cracking, and other forms of distress [12,13,14,15,16,17,18,19,20], their performance depends on factors such as modifier type and dosage, binder–modifier compatibility, and mixing conditions [4,21]. Moreover, high material cost, increased energy demand, storage stability problems, and phase separation may limit the practical and environmental benefits of some conventional modification methods [4,7,8,19,22].

Growing concern over resource consumption and carbon emissions in road construction has therefore increased interest in renewable and waste-derived materials for bitumen modification [7,8,22,23,24]. Among these materials, mineral fillers are particularly attractive because their physicochemical characteristics, particle morphology, and dosage can substantially influence the rheological, mechanical, and adhesive properties of bituminous binders and mastics [25,26]. Recent studies further demonstrate that filler type and content can affect deformation resistance, fatigue behavior, asphalt–filler interactions, and adhesion [25,26]. Industrial by-products such as glass powder, ceramic waste powder, fly ash, steel slag, and red mud have consequently been investigated as mineral-based modifiers or fillers in bituminous materials [27,28,29,30,31,32,33,34,35,36,37,38]. Nevertheless, the performance of these materials depends strongly on their characteristics and interaction with bitumen [39,40,41], and the behavior of high-silica waste materials remains of particular interest [42,43,44].

During glass production, quartz sand is subjected to crushing and wet-sieving processes. Following the separation of the components required for glass manufacturing, waste quartz sand (WQS) sludge containing solid particles smaller than 300 μm is generated [45]. Although WQS is generally considered a non-hazardous industrial waste, its uncontrolled storage and disposal can create environmental concerns, particularly during dry periods when fine particles may become airborne [46,47]. The storage and transportation of substantial quantities of WQS generated by glass manufacturing facilities may also create additional economic burdens [45]. Therefore, the valorization of WQS in value-added applications represents a potential approach to reducing waste accumulation and improving resource utilization.

Despite increasing interest in the beneficial utilization of WQS, its application in pavement materials remains relatively limited. In contrast, other industrial mineral wastes such as coal gangue, mine tailings, copper tailings, and iron tailings have increasingly been investigated as alternative fillers or modifiers in asphalt-related applications [29,38,39,44]. WQS, by comparison, is characterized by a predominantly silica-rich composition and fine particle characteristics, providing a distinct material profile for evaluation in bituminous systems. Within the existing WQS-specific literature, however, most studies have focused on soil and road subbase stabilization [46,48]. Karakılçık and Türkmen [45] investigated WQS as a filler material in combination with cement raw materials and fly ash-treated clay and reported favorable results. In asphalt mixtures, Taciroğlu et al. [49] evaluated WQS as a mineral filler as a partial replacement for limestone-based filler and reported improved rutting resistance at a 50% replacement level. However, the direct use of WQS as a modifier of bituminous binders and its effects on binder-level physical and rheological properties, aging behavior, storage stability, and adhesion performance remain insufficiently investigated.

Accordingly, this study investigated the potential use of finely processed WQS as a mineral-based modifier for B50/70 bitumen. WQS was incorporated at 0%, 1%, 2%, 3%, and 4% by weight, and the resulting binders were evaluated in terms of conventional physical properties, storage stability, high-, intermediate-, and low-temperature rheological behavior, short- and long-term aging response, and aggregate–binder adhesion. In addition, a screening-level environmental and economic assessment was conducted to examine the potential benefits associated with WQS utilization. Through this combined evaluation, the study aimed to determine the influence of WQS content on binder performance and identify a suitable modification level among the investigated dosages.

**Figure 1:**
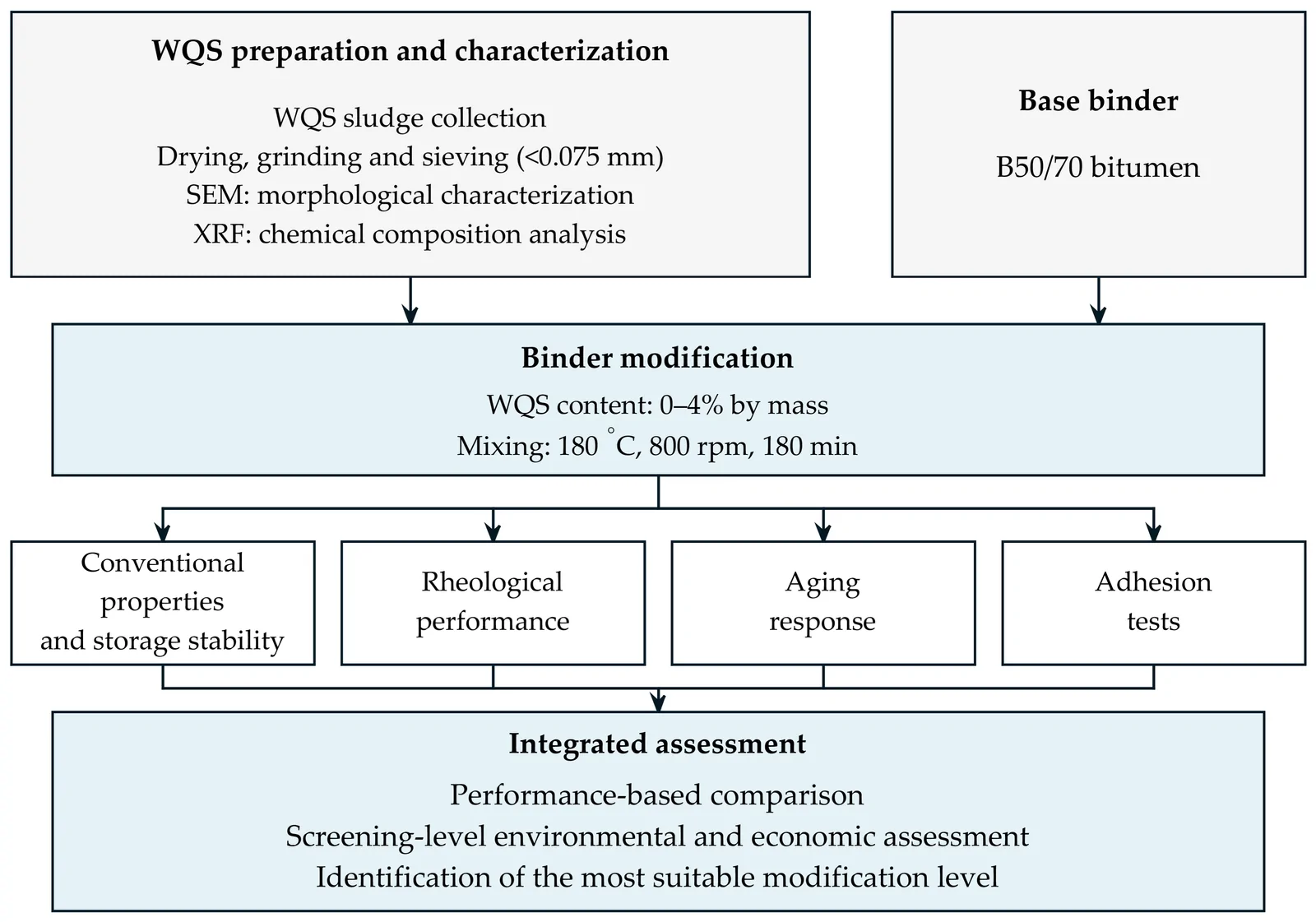
Technical roadmap of the experimental program and integrated assessment of WQS-modified bituminous binders.

The overall experimental framework adopted in this study is summarized in Figure 1.

## 2. Materials and Methods

### 2.1. Materials

#### 2.1.1. Bitumen

The base bitumen used in this study was produced at the Batman Refinery and obtained from Asphalt Plant No. 3 (İskenderun) of the 5th Regional Directorate of the General Directorate of Highways, Mersin, Türkiye. The main physical properties of the base bitumen are presented in Table 1.

#### 2.1.2. WQS

**Figure 2:**
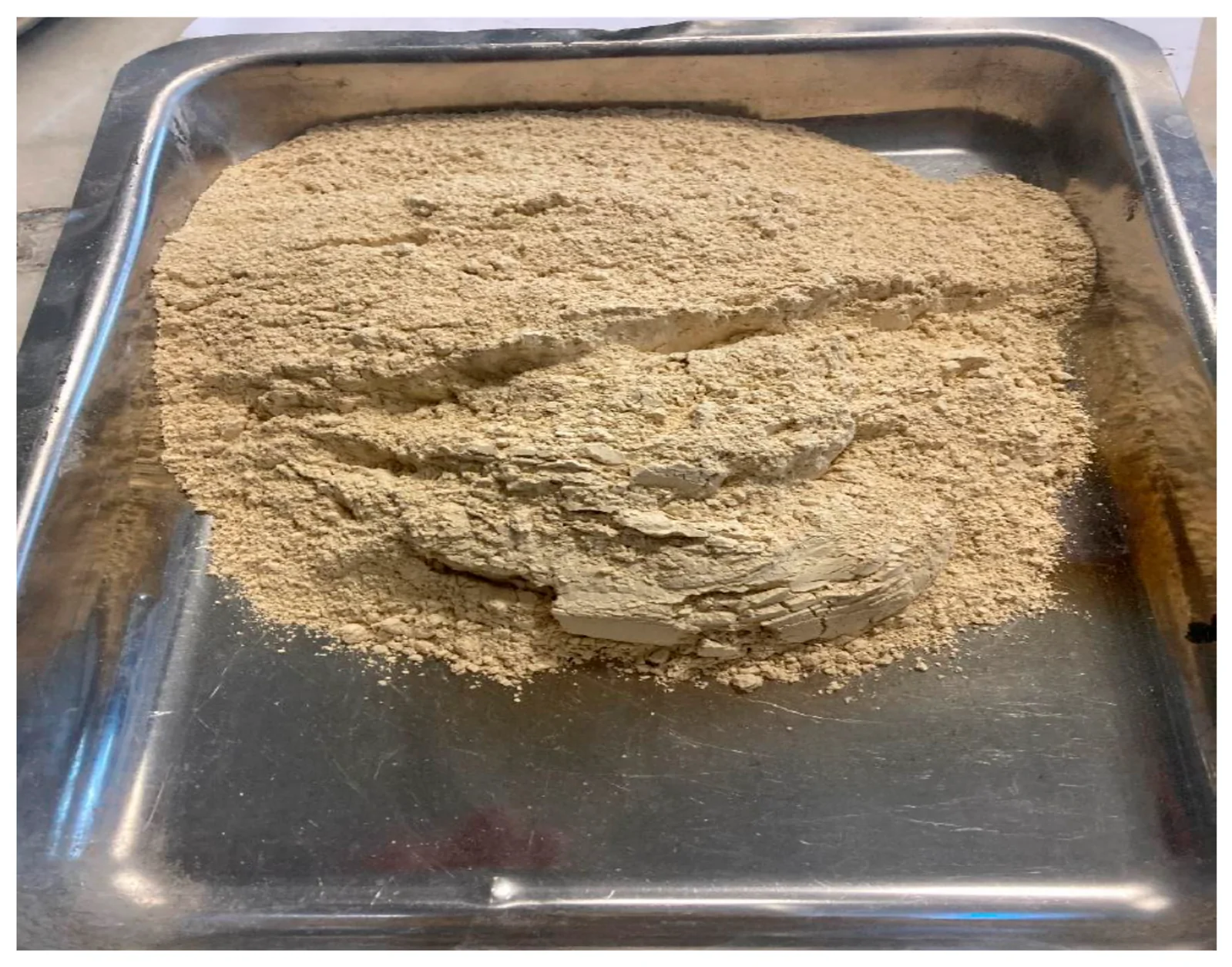
Sample of waste quartz sand.

The waste quartz sand used in this study was obtained as sludge from a glass manufacturing plant in Mersin, Türkiye. In a previous study, Taciroğlu et al. [49] initially dried the WQS sludge in an oven at 105 °C for 24 h. The hardened sludge was then mechanically broken down and subsequently ground in a vibrating disc mill for 1 min to obtain a fine powder. The ground material was then sieved using an ASTM standard sieve series through No. 40 (0.425 mm), No. 80 (0.180 mm), and No. 200 (0.075 mm) sieves. The sieve analysis showed that 100% of the WQS passed the No. 40 sieve, 99.6% passed the No. 80 sieve, and 96% passed the No. 200 sieve. These results indicated that WQS consisted predominantly of filler-sized particles [49]. The same drying and grinding procedures were applied in the present study. The ground material was subsequently sieved through a No. 200 sieve, and the filler-sized material passing this sieve was used as a bitumen modifier (Figure 2).

**Figure 3:**
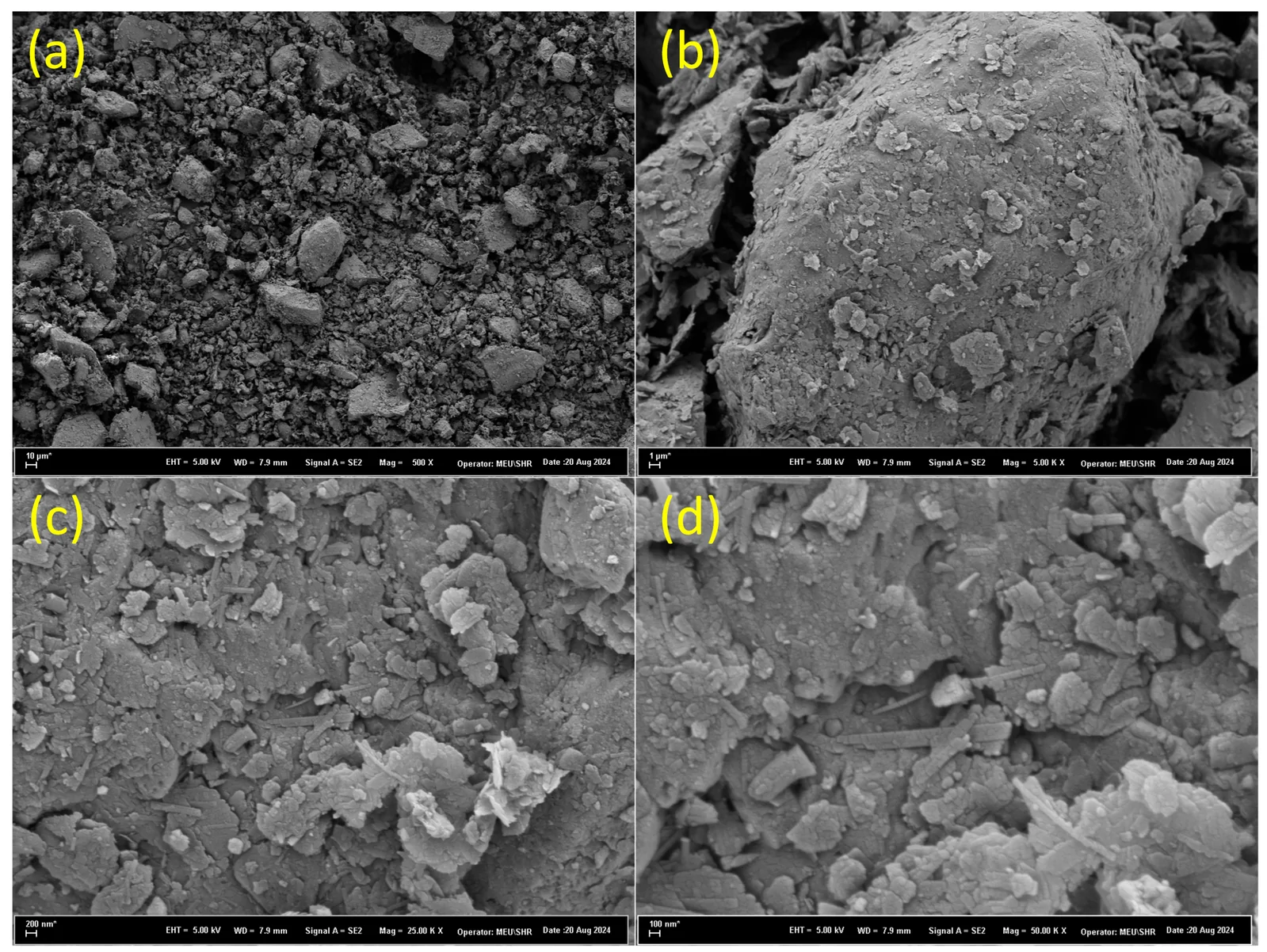
SEM images of WQS at different magnifications: (**a**) 500×, (**b**) 5k×, (**c**) 25k×, and (**d**) 50k×.

The WQS samples were examined using scanning electron microscopy (SEM) to characterize their surface topography. The SEM images presented in Figure 3 show that WQS consists of irregularly shaped and angular particles with varying particle sizes. The low-magnification images reveal the coexistence of fine and coarse particles, with the fine particles exhibiting a distribution that could allow them to fill the voids between the larger particles (Figure 3a,b). At higher magnifications, the particle surfaces appear distinctly rough and irregular, with micro-protrusions and fracture marks observed (Figure 3c,d). This morphological structure is a natural consequence of the crushing and screening processes used in glass production and contributes to an increase in the particles’ specific surface area. From the perspective of bitumen modification, the angular and rough surface morphology may contribute to physical interactions between the bitumen and WQS particles; however, direct mechanical interlocking within the binder matrix cannot be confirmed from the present SEM observations. In addition, the presence of fine particles may contribute to the formation of a denser bitumen matrix. Therefore, the SEM analysis indicates that WQS possesses morphological characteristics that may influence the physical response of the modified binder. However, the underlying interaction mechanisms cannot be confirmed from the SEM images alone.

To determine the chemical composition of WQS, X-ray fluorescence (XRF) analysis was performed on the WQS samples at the Advanced Technology Education, Research and Application Center (MEITAM), Mersin University, Türkiye. The results are presented in Table 2. The XRF analysis showed that WQS predominantly consists of SiO_2_, followed by Al_2_O_3_, K_2_O, CaO, MgO, and Fe_2_O_3_. These results confirm that WQS is a mineral-based industrial waste with a high silica content and a predominantly inorganic composition. From the perspective of bitumen modification, the mineral composition of WQS may influence its interaction with the bitumen matrix. Recent studies have demonstrated that asphalt–mineral interactions are influenced by both oxide composition and surface characteristics, with different mineral oxides exhibiting different interfacial affinities toward bituminous components [50,51,52,53]. In particular, the presence and relative proportions of oxides such as CaO and MgO may influence asphalt–mineral interfacial behavior [50,51,52,53]. In addition, recent studies on nano-silica-modified bituminous binders have reported changes in binder consistency and rheological behavior, including increased stiffness and improved high-temperature performance at appropriate modification levels [54,55,56,57]. Although WQS differs from engineered nano-silica in particle size and material characteristics, its high SiO_2_ content and fine particle characteristics may contribute to the changes observed in the physical and rheological properties of the WQS-modified binders. However, the present XRF analysis does not provide direct evidence of specific chemical interactions between WQS and bitumen.

When the SEM and XRF analyses are considered together, the irregular and rough surface morphology of WQS is consistent with its mineral composition and high silica content. The fine particle size and irregular, rough surface morphology suggest that WQS has the potential to act as an effective micro-filler within the bitumen matrix, while its silicate-based mineral composition may contribute to increased binder stiffness and resistance to deformation, particularly at elevated temperatures. Therefore, WQS should not be considered solely as a waste material from an environmental perspective; it may also be regarded as a sustainable mineral additive with potential for use in bitumen modification.

### 2.2. Methods

Following the incorporation of WQS into the base bitumen, the resulting binders were characterized through a comprehensive experimental program that evaluated their conventional physical properties, rheological behavior at high, intermediate, and low temperatures, aging characteristics, and binder–aggregate adhesion. These tests were selected to evaluate the effects of WQS modification on the engineering performance of the binders under different service and production conditions. Binder tests were performed in the laboratories of the Republic of Türkiye General Directorate of Highways, 5th Regional Directorate/Mersin.

#### 2.2.1. Preparation of Modified Bitumen Samples

Modified bituminous binders were prepared by incorporating 1%, 2%, 3%, and 4% WQS by weight into B50/70 penetration-grade base bitumen. To ensure the homogeneous dispersion of WQS within the base bitumen matrix, the bitumen–WQS mixtures were blended at 180 °C using a mechanical mixer at 800 rpm for 180 min. After mixing, the modified bitumen samples were poured into sample containers.

#### 2.2.2. Conventional Physical Properties

The conventional physical properties of the binders were evaluated through penetration, softening point, flash point, ductility, and storage stability tests. Penetration was determined in accordance with TS EN 1426 [58] at 25 °C under a 100 g load for 5 s, while the softening point was measured using the ring-and-ball method according to TS EN 1427 [59]. Flash point and ductility tests were conducted in accordance with TS EN ISO 2592 [60] and TS EN 13589 [61], respectively. Storage stability was evaluated in accordance with TS EN 13399 [62] by conditioning the samples in aluminum tubes at 180 °C for 3 days and comparing the penetration and softening point values from the upper and lower sections of the tubes [11].

#### 2.2.3. Rheological Properties

The rheological behavior of the binders was evaluated using a dynamic shear rheometer (DSR) and a bending beam rheometer (BBR). The DSR temperature sweep tests were performed in accordance with TS EN 14770 [63] to characterize the viscoelastic behavior of the binders at intermediate and high temperatures. The test temperatures were selected with reference to the Superpave performance grading (PG) temperature framework. The tests were conducted in strain-controlled mode at a constant angular frequency of 10 rad/s using parallel-plate geometries. For the unaged and short-term-aged binders, a 25 mm parallel plate with a 1 mm gap was used, with shear strain amplitudes of 12% and 10%, respectively. The high-temperature tests were initiated at 52 °C and continued at discrete 6 °C intervals, with the final test temperature depending on the response of each binder. For the long-term-aged (RTFO+PAV) binders, an 8 mm parallel plate with a 2 mm gap was used, and the shear strain amplitude was set to 1%. The intermediate-temperature tests were initiated at 25 °C and continued toward lower temperatures at discrete 3 °C intervals, with the final test temperature varying among the binders. Similar discrete-temperature approaches, using 6 °C intervals for unaged and short-term-aged binders and 3 °C intervals for long-term-aged binders, have also been adopted in recent DSR-based rheological studies [64,65]. Within each aging condition, all binders were tested using the same discrete temperature increments and sequence up to the applicable final test temperature. The reported values correspond to directly measured experimental data, and no intermediate values were generated by interpolation.

The complex shear modulus (G*) and phase angle (δ) were obtained as the principal DSR parameters. G* represents the overall resistance of the binder to shear deformation, whereas δ reflects the relative contribution of elastic and viscous behavior [66,67]. The rutting parameter (G*/sin(δ)) was used to evaluate the high-temperature rutting-related rheological response of the unaged and short-term-aged binders, with higher values indicating a more favorable high-temperature rheological response [68,69]. For the long-term-aged binders, the fatigue parameter (G*·sin(δ)) was used to evaluate the fatigue-related rheological response at intermediate temperatures, with lower values indicating a more favorable fatigue-related rheological response.

The low-temperature rheological properties of the binders were evaluated using the BBR test. At sufficiently low temperatures, asphalt binders become increasingly stiff and more susceptible to thermally induced tensile stresses, which may promote thermal cracking [70]. The BBR test was therefore used to determine the creep stiffness (S) and m-value of the binders. According to the commonly used criteria specified in ASTM D6648 [71], the creep stiffness at 60 s should not exceed 300 MPa, while the m-value should be at least 0.300.

#### 2.2.4. Aging Properties

Aging is one of the key factors affecting the long-term performance of asphalt binders. During asphalt production, transportation, mixing, and compaction, binders are exposed to high temperatures and atmospheric oxygen, resulting in short-term oxidative aging [64]. In the laboratory, this aging stage was simulated using the Rolling Thin Film Oven (RTFO) procedure in accordance with TS EN 12607-1 [72]. The binder samples were conditioned at 163 °C for 85 min while the bottles were continuously rotated about their vertical axes. At the end of the conditioning period, the samples were weighed, and the mass change was calculated using Equation (1) [72]:

(1) $$∆m\left(\%\right)=\frac{m_{2}-m_{1}}{m_{1}-m_{0}}\;\times \;100$$

where (*m*_0_) is the mass of the empty glass bottle, (*m*_1_) is the mass of the bottle and binder before RTFO conditioning, and (*m*_2_) is the mass of the bottle and binder after RTFO conditioning. A negative value indicates mass loss, whereas a positive value indicates mass gain. The binders obtained after RTFO conditioning were subsequently used to evaluate changes in binder properties associated with short-term aging.

Long-term aging was simulated by subjecting the RTFO-aged binders to Pressure Aging Vessel (PAV) conditioning in accordance with TS EN 14769 [73]. The binders were aged for 20 h at 100 °C under an air pressure of 2.1 ± 0.1 MPa. The resulting long-term-aged binders were subsequently characterized to evaluate the effect of WQS modification on aging-related rheological performance.

#### 2.2.5. Aggregate–Binder Adhesion Properties

The durability of the aggregate–binder interface is an important factor governing the moisture resistance of asphalt mixtures. Moisture infiltration and environmental exposure may weaken the adhesion between the binder and aggregate surface, promoting stripping and other forms of moisture-induced damage [74]. Therefore, the Nicholson stripping and Vialit adhesion tests were performed to evaluate the adhesion performance of the WQS-modified binders. The limestone aggregates used in the adhesion tests had a Los Angeles abrasion loss of 26%, a freeze–thaw loss of 7%, and a flakiness index of 13.6% [11].

The Nicholson stripping test was conducted in accordance with TS EN 12697-11 [75]. Aggregate specimens coated with binder were immersed in distilled water at 19 ± 1 °C for 48 ± 1 h, and the stripping behavior was evaluated by visually determining the amount of aggregate surface remaining coated with binder after conditioning. The test results were expressed as the percentage of the aggregate surface remaining coated with bitumen (%). The Vialit adhesion test was performed in accordance with TS EN 12272-3 [76]. The bituminous binder was uniformly applied to the plate surface, after which the aggregates were placed on the binder-coated plate and compacted using a rubber-tired roller. The prepared plates were then conditioned under controlled conditions. Following impact loading, the detached aggregate particles were counted, and the percentage of aggregate particles remaining attached to the plate was calculated and reported as the adhesion rate (%).

## 3. Results and Discussion

### 3.1. Conventional Binder Tests

The results of the conventional binder tests indicate that increasing the WQS content resulted in noticeable changes in the physical properties of the binders. The most pronounced change was observed in the penetration values, which decreased continuously as WQS content increased. Compared with the neat bitumen, the penetration values decreased by 17.5%, 37.4%, 42.0%, and 42.7% for the binders containing 1%, 2%, 3%, and 4% WQS, respectively. This decrease indicates that WQS increased the stiffness of the bitumen matrix and resulted in a harder binder consistency. The reduction in penetration may be attributed primarily to the filler effect of the fine-grained, silica-rich WQS particles within the bitumen matrix, which may restrict the mobility of the bituminous phase. Similarly, the specific gravity exhibited a limited but consistent increase with increasing WQS content, ranging from approximately 0.2% at 1% WQS to 2.6% at 4% WQS compared with neat bitumen. This trend is consistent with the incorporation of the quartz-based mineral additive into the binder system and the corresponding increase in specific gravity. The softening point remained nearly unchanged at 1% WQS, with a slight decrease of approximately 0.3%, whereas increases of 4.2%, 5.7%, and 7.6% were observed at WQS contents of 2%, 3%, and 4%, respectively. These increases at higher WQS contents indicate an improvement in the high-temperature response of the modified binders and suggest that WQS-modified binders may become more resistant to flow at elevated temperatures. In contrast, the flash point exhibited only minor variations with WQS incorporation. Relative to the neat bitumen, no change was observed at 1% WQS, while reductions of approximately 5.1%, 5.1%, and 3.4% were obtained at 2%, 3%, and 4% WQS, respectively. Despite these changes, all modified binders remained above the minimum safety limit specified in the Road Technical Specifications (2023) [77], indicating that WQS incorporation does not adversely affect the safety of the binders during production and application. The ductility results indicate that the binder’s ductility was maintained at WQS contents of up to 2%, with reductions of approximately 22.0% and 46.8% at 3% and 4% WQS, respectively. This reduction suggests that higher WQS contents decrease the deformation capacity of the binder and promote a more brittle response. This behavior may be attributed to the increased mineral content, which can reduce the continuity of the bituminous phase and consequently limit the elongation capacity of the binder. The results of the conventional binder tests conducted on the neat and WQS-modified binders are presented in Table 3.

Overall, WQS modification increased the stiffness of the bitumen and improved its high-temperature response, while reducing ductility at higher modification levels. In particular, the simultaneous decrease in penetration and increase in softening point at WQS contents of 2–4% suggest that WQS-modified binders may exhibit greater resistance to flow under high-temperature conditions. However, the increased stiffness and the pronounced loss of ductility at higher WQS contents highlight the importance of determining an appropriate WQS content. This behavior may be associated with the mineral characteristics of WQS, which contains approximately 68% SiO_2_ according to the XRF analysis. The rigid mineral particles dispersed within the bitumen matrix may contribute to restricting deformation and increasing resistance to flow at elevated temperatures. The silica-rich mineral composition and fine-particle characteristics of WQS may contribute to the observed decrease in penetration and increase in softening point; however, the underlying interaction mechanism cannot be confirmed from conventional binder tests alone.

### 3.2. Storage Stability Tests

The neat and modified bitumen samples stored in tubes were subjected to softening point and penetration tests at the end of the storage period. The storage stability results showed that the softening point values measured at the top and bottom sections of the tubes after storage were generally similar for all binders. The difference in softening point (ΔSP) between the top and bottom sections ranged from 0.1 to 0.6 °C and corresponded to less than 1% of the mean softening point in all cases. These results indicate that no pronounced phase separation occurred during storage. The highest softening point was obtained for the 4% WQS-modified binder, consistent with the conventional binder test results. However, this binder also exhibited the highest ΔSP value, whereas the neat and 1% WQS-modified binders showed the smallest difference. Compared with the values measured before storage, the mean softening point increased by approximately 2.2–6.1% across the investigated binders. Although the differences between the upper and lower sections were small for all binders, the results suggest slightly greater variation between the upper and lower sections at higher WQS contents.

The penetration results obtained after storage showed a different trend. Compared with the neat bitumen, the average penetration values of the 1% and 2% WQS-modified binders decreased by approximately 1.8% and 6.7%, respectively, indicating a moderate increase in consistency. In contrast, the average penetration values of the 3% and 4% WQS-modified binders increased by approximately 13.2% and 30.7%, respectively. The differences between the top and bottom penetration values remained relatively limited, corresponding to approximately 2.3%, 3.6%, 1.0%, and 5.7% for the binders containing 1%, 2%, 3%, and 4% WQS, respectively. This variation may be related to changes in the physical interactions between the WQS particles and the bitumen during storage. Nevertheless, the relatively small differences between the upper and lower sections indicate that the applied storage conditions did not lead to pronounced non-uniformity in penetration. The detailed storage stability results are presented in Table 4.

Overall, the small differences in softening point between the top and bottom sections indicate satisfactory storage stability for the investigated WQS-modified binders. The slightly higher ΔSP and difference in penetration observed at 4% WQS suggest that increasing the WQS content may have a minor effect on the uniformity of the modified binder during storage. However, the observed differences remained limited, and the storage stability results indicated no pronounced phase separation. The variation in penetration values after storage further indicates that the effect of WQS content on binder properties cannot be fully described using conventional tests alone. Therefore, the rheological behavior of the WQS-modified binders was further evaluated using DSR and BBR tests.

### 3.3. Rolling Thin Film Oven (RTFO) Tests

The mass change, softening point, and penetration results obtained after the RTFO test were evaluated following short-term aging in accordance with TS EN 12607-1. The mass change values of all binders were found to be very small, ranging from −0.04% to −0.02%. These limited mass changes indicate that the incorporation of WQS did not have a significant adverse effect on the loss of volatile components from the binders. This result demonstrates that the WQS-modified binders possess adequate thermal stability at the production and mixing temperatures.

Compared with the neat bitumen, a slight variation in mass change was observed for the WQS-modified binders, with the 2% WQS-modified binder exhibiting the smallest mass loss and the 3% WQS-modified binder showing the highest mass loss. Nevertheless, the fact that the mass changes of all samples remained very small indicates that WQS incorporation did not substantially increase the loss of volatile components during short-term aging. The penetration results obtained after RTFO showed that all WQS-modified binders exhibited lower penetration values than the neat bitumen. Compared with the neat bitumen, the penetration values decreased by 55.3%, 69.4%, 64.2%, and 58.0% for the binders containing 1%, 2%, 3%, and 4% WQS, respectively. The most pronounced reduction was observed for the 2% WQS-modified binder. This decrease indicates that WQS modification increased binder stiffness and resistance to plastic deformation following short-term aging. The softening point results further support the penetration findings. Compared with the neat bitumen, the softening point increased by 16.1%, 26.1%, 37.4%, and 42.7% for the 1%, 2%, 3%, and 4% WQS-modified binders, respectively. The softening point difference also increased progressively with increasing WQS content. Accordingly, the increase in the softening point and the softening point difference with increasing WQS content indicate that WQS incorporation enhanced the binder’s resistance to flow at elevated temperatures and promoted structural hardening following short-term aging.

The penetration ratio (PR) results showed that the neat bitumen exhibited the highest PR value, whereas the WQS-modified binders exhibited considerably lower values. Compared with the neat bitumen, PR decreased by 45.8%, 51.1%, 38.2%, and 26.7% for the binders containing 1%, 2%, 3%, and 4% WQS, respectively. The lowest PR value was obtained for the 2% WQS-modified binder. This finding indicates that WQS incorporation altered the penetration response of the binders during short-term aging, with the 2% WQS-modified binder showing the greatest degree of penetration reduction relative to its unaged condition. The detailed RTFO results are presented in Table 5.

The RTFO results indicate that WQS incorporation can improve the high-temperature performance of the binders following short-term aging. The decrease in penetration and increase in softening point suggest that the presence of WQS contributed to increased resistance to deformation. In addition, the very small mass change values of all binders indicate that WQS incorporation did not adversely affect the thermal stability of the binders under short-term aging conditions. These findings suggest that WQS may provide a beneficial contribution to the high-temperature performance of bitumen by modifying its physical properties under short-term aging conditions.

### 3.4. DSR Temperature Sweep Tests

In this study, DSR temperature sweep tests were conducted to determine the complex shear modulus (G*) and phase angle (δ) of the neat and modified binder specimens in the unaged, short-term-aged (RTFO-aged) and long-term-aged (RTFO+PAV-aged) conditions. In accordance with the applicable testing standards, specimens with a diameter of 25 mm were prepared for the DSR temperature sweep testing of the unaged and short-term-aged binders, whereas 8 mm-diameter specimens were prepared for the long-term-aged binders.

#### 3.4.1. Temperature Sweep Results of Unaged Binders

**Figure 4:**
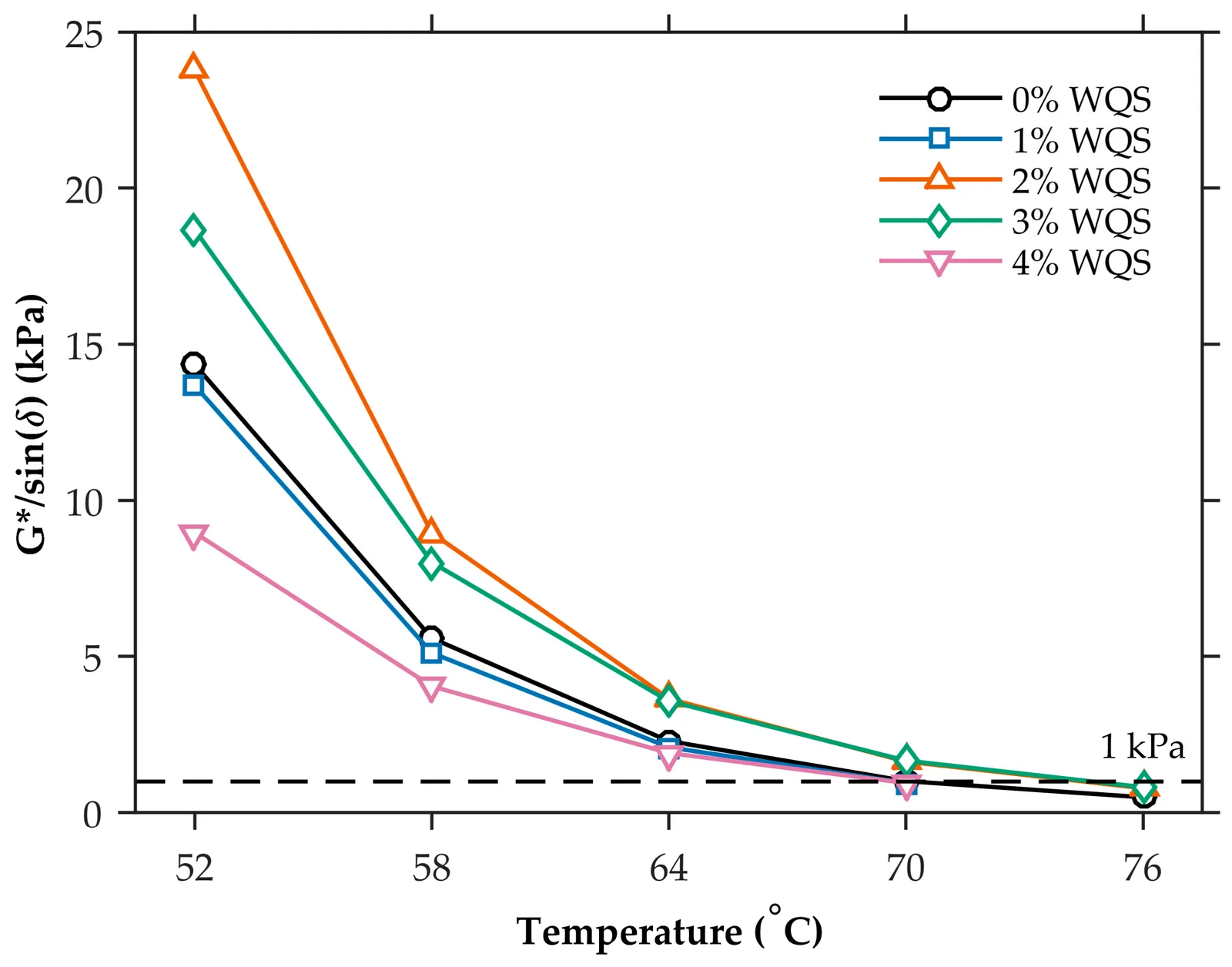
Variation in G*/sin(δ) with temperature for unaged binders.

**Figure 5:**
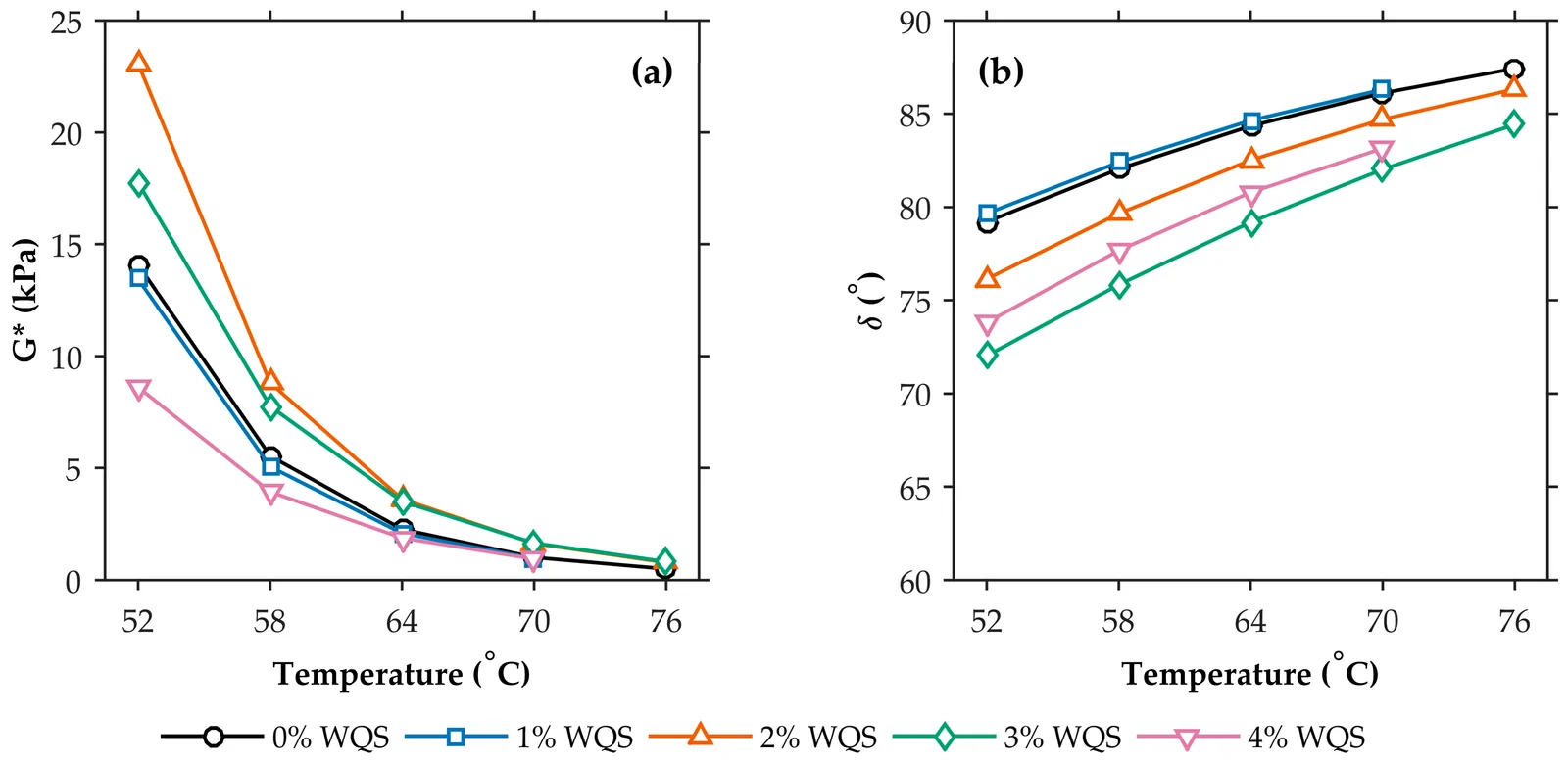
Variation in (**a**) complex shear modulus and (**b**) phase angle with temperature for unaged binders.

The results obtained from the temperature sweep tests conducted on the unaged binders are presented in Figure 4 and Figure 5. As described in Section 2.2.3, the measurements were conducted at discrete 6 °C temperature intervals. According to the Superpave performance grading system, the minimum required value of the G*/sin(δ) parameter for evaluating the rutting-related high-temperature response of unaged binders is 1 kPa. Based on this criterion, among the discrete temperatures investigated, the neat bitumen and the binders containing 2% and 3% WQS satisfied the specification limit up to a highest tested temperature of 76 °C, whereas the binders containing 1% and 4% WQS satisfied the criterion up to a highest tested temperature of 70 °C (Figure 4). Thus, the 2% and 3% WQS-modified binders maintained compliance with the criterion at a tested temperature 6 °C higher than binders containing 1% and 4% WQS. These results indicate that WQS incorporation at 2–3% provided a more favorable high-temperature rheological response, whereas both lower and higher WQS contents provided less favorable high-temperature performance.

The G* values decreased with increasing temperature for all binders, as expected (Figure 5a). This behavior can be attributed to the increasing dominance of the viscous component of bitumen at elevated temperatures, resulting in a reduction in binder stiffness. Nevertheless, the G* values of the binders containing 2% and 3% WQS were higher than those of the neat bitumen over the 52–70 °C temperature range. This finding indicates that WQS incorporation increased the overall stiffness of the binder and improved its high-temperature rheological response. In particular, the 2% WQS-modified binder exhibited the highest complex modulus values over the entire temperature range, suggesting that this dosage may represent a favorable WQS content among the investigated dosages. In contrast, the 1% WQS-modified binder exhibited G* values similar to those of the neat bitumen, indicating a relatively limited effect of the modification. More notably, the 4% WQS-modified binder exhibited the lowest G* values at all temperatures. This finding suggests that increasing the WQS content beyond the more favorable range did not provide a further improvement in binder stiffness. This behavior may be related to differences in particle dispersion and particle–binder interactions at higher WQS contents. The irregular and angular morphology of WQS particles observed in the SEM analysis may also influence their interaction with the bitumen matrix. However, the exact mechanism responsible for the lower G* values at the 4% WQS content cannot be determined solely from the present DSR results.

The δ values increased with increasing temperature for all binders and approached 90° as the failure temperature was approached (Figure 5b). This behavior indicates that the binders gradually deviated from predominantly elastic behavior and exhibited a more viscous character with increasing temperature. Nevertheless, all WQS-modified binders exhibited slightly lower δ values than the neat bitumen. This reduction in phase angle indicates a greater elastic contribution to the overall viscoelastic response of the binder. In particular, the simultaneous occurrence of higher G* and lower δ values for the 2% and 3% WQS-modified binders indicates that these binders may exhibit a more favorable rutting-related rheological response at elevated temperatures.

The observed improvement may be associated with the fine particle size and silica-rich mineral composition of WQS. The WQS particles, containing approximately 68% SiO_2_ and predominantly passing the 0.075 mm sieve, may contribute to the stiffening of the bitumen matrix when incorporated at contents of 2–3%. Their fine mineral structure may enhance the overall stiffness of the binder and thereby contribute to a more favorable high-temperature response. However, the lower G* values observed at the 4% WQS content indicate that increasing the modifier content beyond the more favorable range did not provide a further improvement in rheological performance. The specific contribution of particle dispersion and particle–binder interactions at this dosage cannot be determined from the present results alone.

Overall, the DSR results demonstrate that WQS incorporation at appropriate contents can improve the high-temperature rheological properties of bitumen, whereas further increasing the modifier content does not necessarily provide additional benefits. In particular, the 2% WQS-modified binder exhibited the most favorable performance in terms of both complex shear modulus and the G*/sin(δ) parameter, indicating a more favorable high-temperature and rutting-related rheological response. In contrast, although the 4% WQS-modified binder exhibited a relatively greater elastic contribution, its lower complex modulus resulted in lower overall stiffness and did not translate into an improved high-temperature rheological response.

#### 3.4.2. Temperature Sweep Results After RTFO

**Figure 6:**
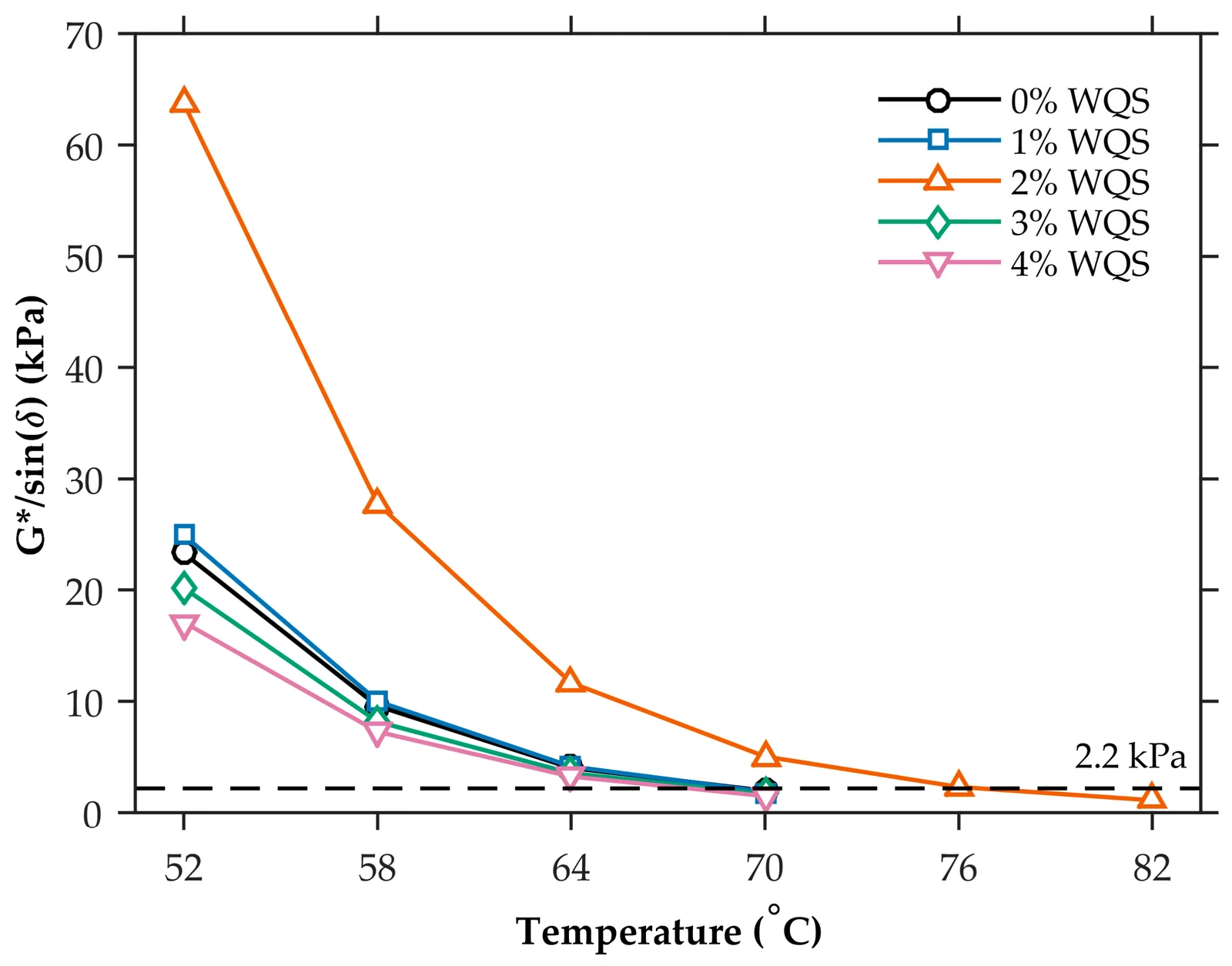
Variation in G*/sin(δ) with temperature for short-term-aged binders.

**Figure 7:**
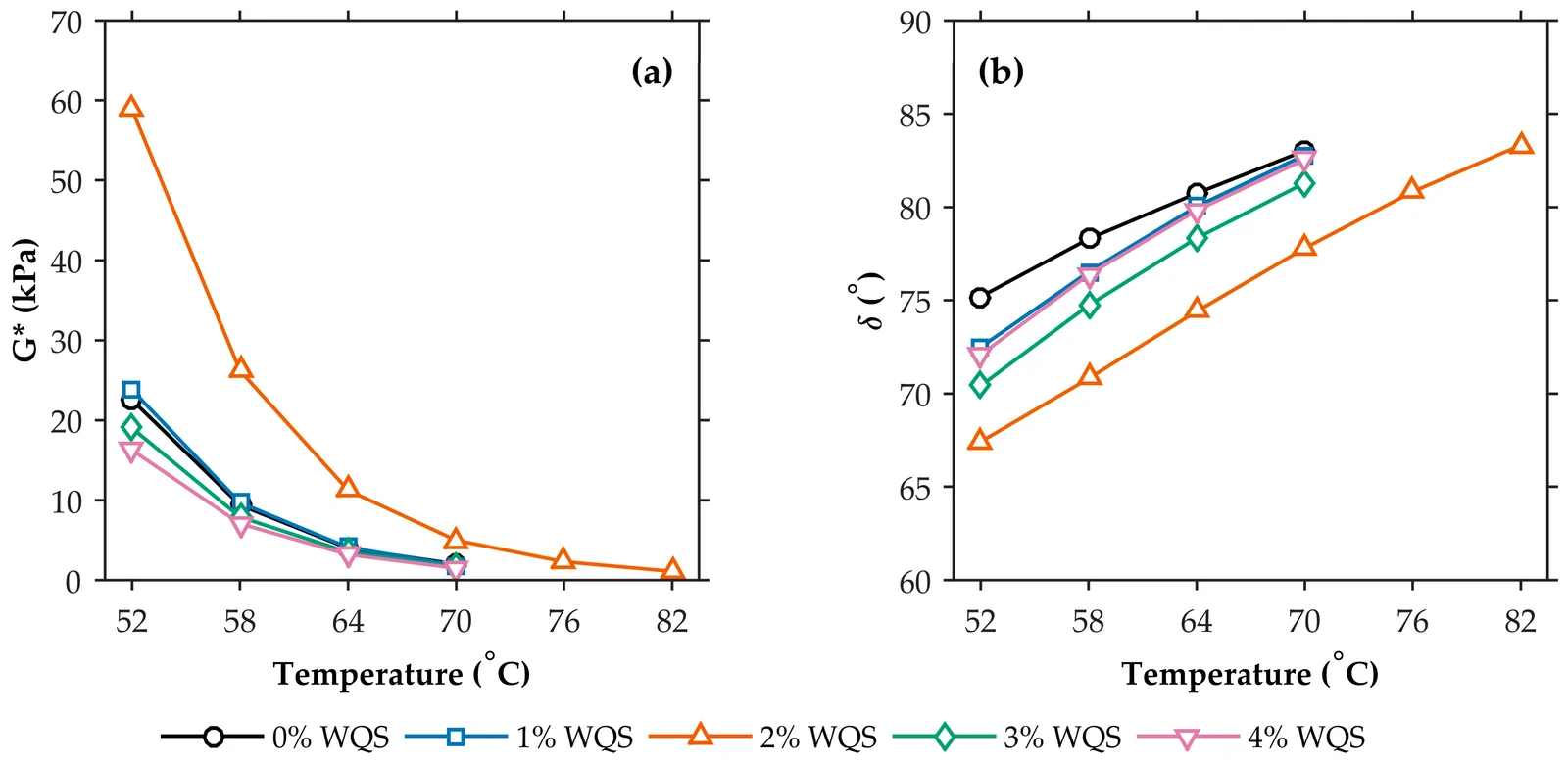
Variation in (**a**) complex shear modulus and (**b**) phase angle with temperature for short-term-aged binders.

The results obtained from the DSR temperature sweep tests conducted after RTFO conditioning to evaluate the high-temperature rheological properties of the binders are presented in Figure 6 and Figure 7. According to the Superpave specification, the G*/sin(δ) parameter of short-term-aged binders should be greater than 2.2 kPa. The test results showed that the neat bitumen and the binders containing 1%, 3%, and 4% WQS satisfied this criterion at 70 °C, whereas the binder containing 2% WQS maintained the specification requirement up to a highest tested temperature of 82 °C (Figure 6). Thus, the 2% WQS-modified binder satisfied the criterion at a tested temperature 12 °C higher than the neat bitumen and the binders containing 1%, 3%, and 4% WQS. These results indicate that the incorporation of 2% WQS substantially improved the high-temperature rheological response of the binder, even after short-term aging.

The G* values decreased with increasing temperature for all binders (Figure 7a). Nevertheless, the 2% WQS-modified binder exhibited distinctly higher G* values than the other binders over the entire temperature range. In particular, the markedly higher G* value observed at 52 °C compared with those of the other binders indicates that WQS incorporation at 2% substantially increased the stiffness of the binder following short-term aging. This behavior may be associated with the stiffening effect of the fine, silica-rich mineral particles within the bitumen matrix and their contribution to the overall rigidity of the binder. In contrast, the 1% WQS-modified binder exhibited G* values similar to those of the neat bitumen, indicating that the lower WQS content had a relatively limited effect on the rheological properties. The 3% and 4% WQS-modified binders, on the other hand, exhibited lower G* values than those of the neat bitumen. This finding suggests that increasing the WQS content beyond the more favorable range did not provide a further increase in binder stiffness. This behavior may be associated with differences in particle dispersion and particle–binder interactions at higher WQS contents. The irregular particle morphology observed in the SEM analysis may also influence the interaction of WQS particles with the bitumen matrix. However, the specific mechanism responsible for the lower G* values at higher WQS contents cannot be determined solely from the present DSR results.

The lowest δ values at 52, 58, 64, and 70 °C were obtained for the 2% WQS-modified binder (Figure 7b). Lower δ values indicate a greater elastic contribution to the overall viscoelastic response of the binder. Accordingly, the simultaneous occurrence of the highest G* and lowest δ values for the 2% WQS-modified binder indicates that this binder exhibited the most favorable high-temperature and rutting-related rheological response among the investigated binders.

#### 3.4.3. DSR Temperature Sweep Results After RTFO+PAV

**Figure 8:**
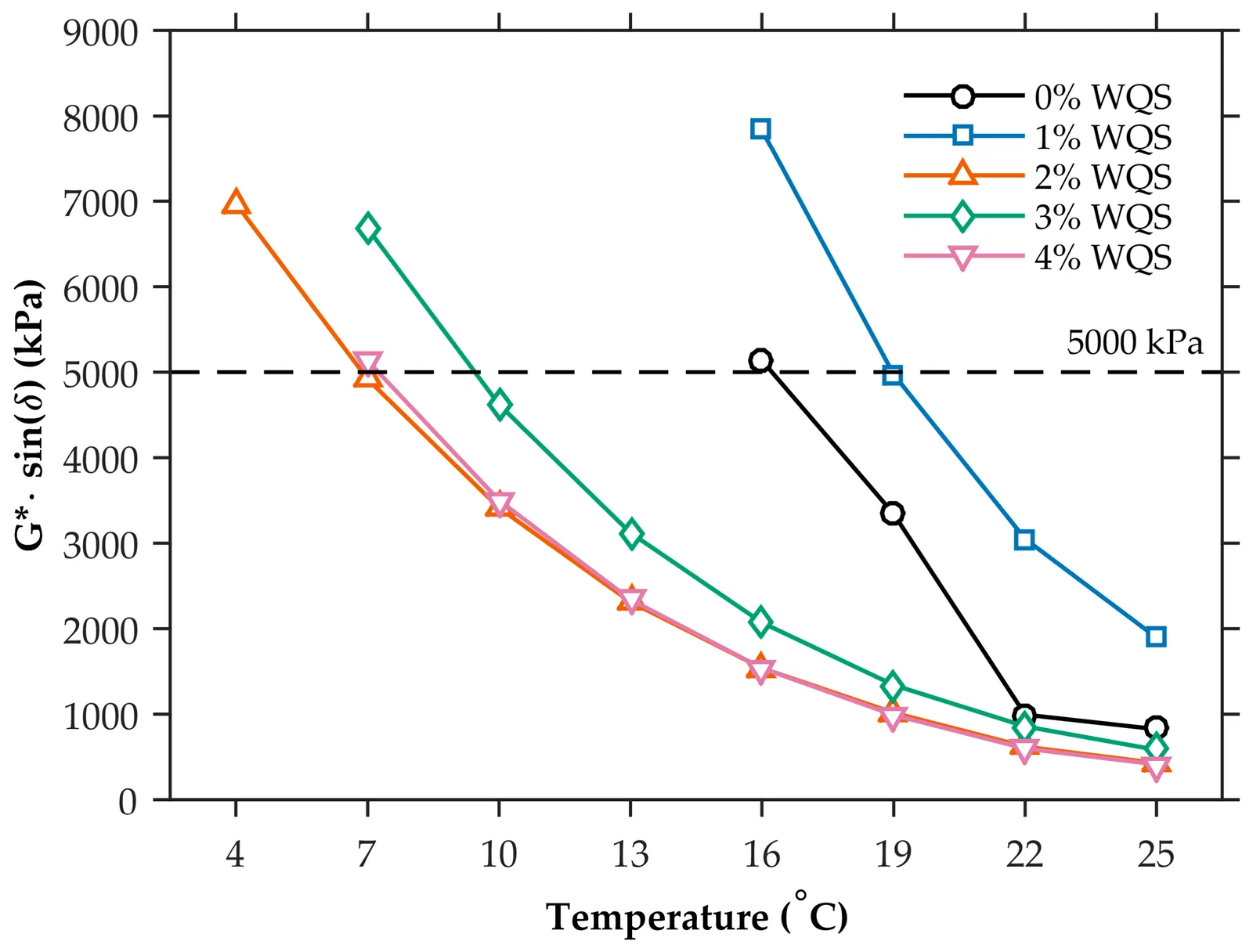
Variation in G*·sin(δ) with temperature for long-term-aged binders.

**Figure 9:**
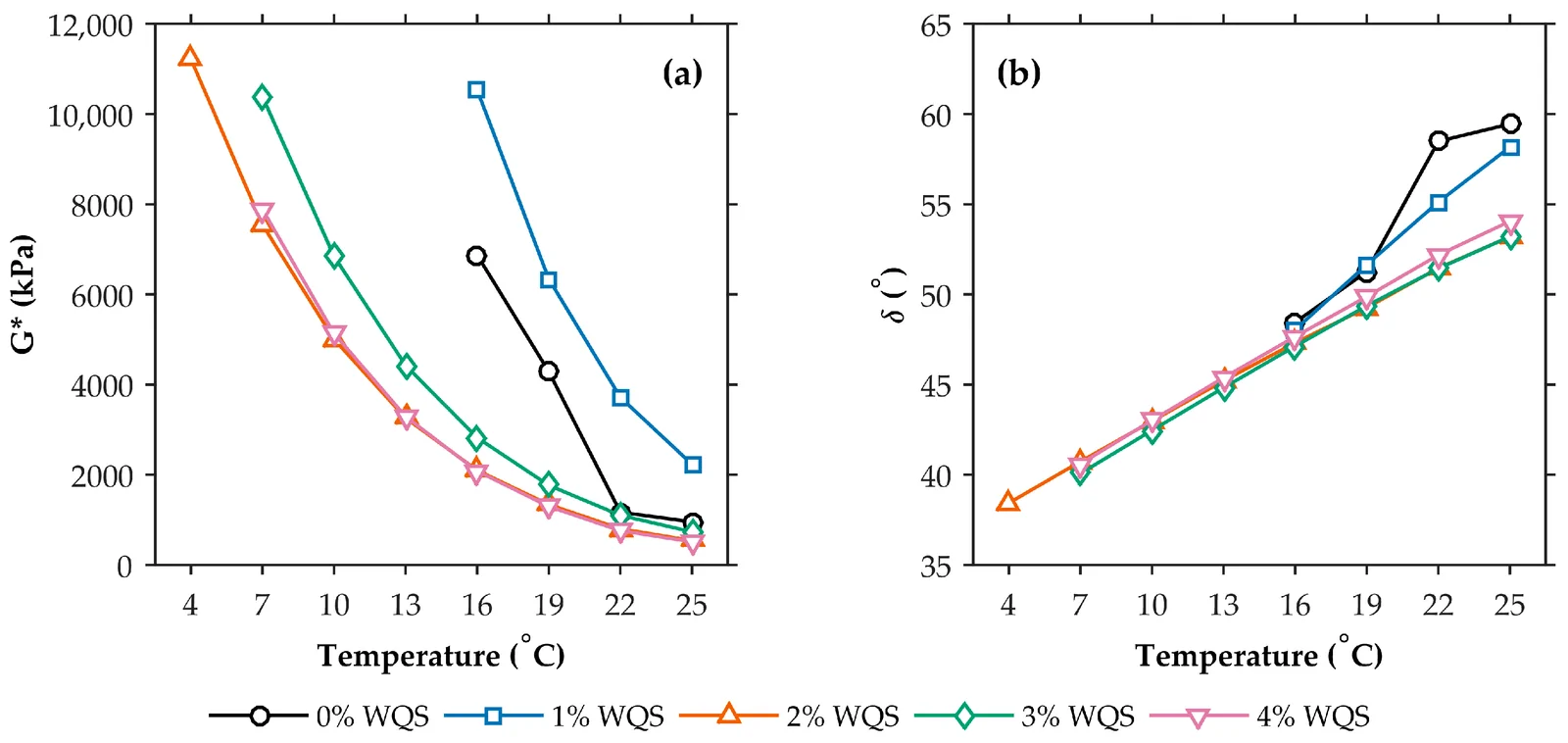
Variation in (**a**) complex shear modulus and (**b**) phase angle with temperature for long-term-aged binders.

The DSR temperature sweep tests were conducted after RTFO and PAV conditioning to evaluate the fatigue-related rheological response of the binders. The initial test temperature was set at 25 °C, and measurements were subsequently conducted at discrete 3 °C intervals. The results are presented in Figure 8 and Figure 9. According to the Superpave performance criteria, the fatigue parameter, G*·sin(δ), of long-term-aged binders should not exceed 5000 kPa. Based on this criterion, among the discrete temperatures investigated, the lowest tested temperature at which the G*·sin(δ) criterion was satisfied was 16 °C for the neat bitumen and the 1% WQS-modified binder, 7 °C for the 3% and 4% WQS-modified binders, and 4 °C for the 2% WQS-modified binder (Figure 8). Thus, compared with the neat bitumen, the 3% and 4% WQS-modified binders maintained compliance with the criterion at a tested temperature 9 °C lower, whereas the 2% WQS-modified binder maintained compliance at a tested temperature 12 °C lower. In addition, the 2% WQS-modified binder satisfied the criterion at a tested temperature 3 °C lower than the 3% and 4% WQS-modified binders. Satisfaction of the G*·sin(δ) ≤ 5000 kPa criterion at a lower tested temperature indicates that the binder may exhibit a more favorable fatigue-related rheological response. From this perspective, WQS incorporation improved the fatigue-related response of the binders after long-term aging, with the most pronounced improvement observed for the 2% WQS-modified binder. The 3% and 4% WQS-modified binders also maintained compliance with the criterion at lower tested temperatures than the neat bitumen, although their performance was less favorable than that of the 2% WQS-modified binder. These comparisons are based on the directly measured discrete temperature points and do not represent interpolated continuous critical temperatures.

The 1% WQS-modified binder exhibited the highest G* values over the 16–25 °C temperature range, whereas the 2%, 3%, and 4% WQS-modified binders exhibited lower G* values than those of the neat bitumen (Figure 9a). Although a lower G* value generally indicates lower stiffness, a higher G* value is not necessarily advantageous for fatigue performance after PAV conditioning, since excessive stiffening may increase the susceptibility of aged binders to brittle behavior and fatigue cracking. Therefore, the lower G* value observed for the 2% WQS-modified binder, together with its lower G*·sin(δ) value, may indicate a more favorable balance between stiffness and fatigue-related rheological response. The phase angle (δ) results showed that the WQS-modified binders exhibited lower phase angle values than those of the neat bitumen at the corresponding tested temperatures (Figure 9b). This reduction indicates a greater elastic contribution to the overall viscoelastic response of the binders. In particular, the combination of lower δ and lower G*·sin(δ) observed for the 2% WQS-modified binder further supports its favorable fatigue-related response following long-term aging.

The observed rheological behavior may be associated with the fine particle size and high SiO_2_ content of WQS. The silica-rich mineral particles may contribute to the overall viscoelastic response of the bitumen matrix and modify its response to aging-induced changes. However, the specific mechanisms responsible for these effects cannot be determined solely from the present DSR results. Similarly, the lower G* values observed at higher WQS contents may be related to differences in particle dispersion and particle–binder interactions, although the dispersion state of WQS within the modified binders was not directly investigated. The irregular and angular particle morphology observed in the SEM analysis and the high silica content identified by XRF provide supporting information for interpreting these results, but do not directly establish the underlying dispersion mechanism.

The DSR temperature sweep results indicate that the effect of WQS on binder rheological performance is strongly dependent on the modification level. Among the investigated dosages, 2% WQS exhibited the most favorable overall performance, particularly in terms of the balance among fatigue-related behavior, stiffness, and elastic response after long-term aging. Meanwhile, 3% and 4% WQS also exhibited a more favorable fatigue-related rheological response, as they maintained compliance with the criterion at tested temperatures 9 °C lower than that of the neat bitumen. These findings suggest that WQS has potential as a sustainable mineral-based modifier for bitumen, although the most favorable dosage may vary depending on the specific performance criterion considered.

### 3.5. BBR Tests

**Figure 10:**
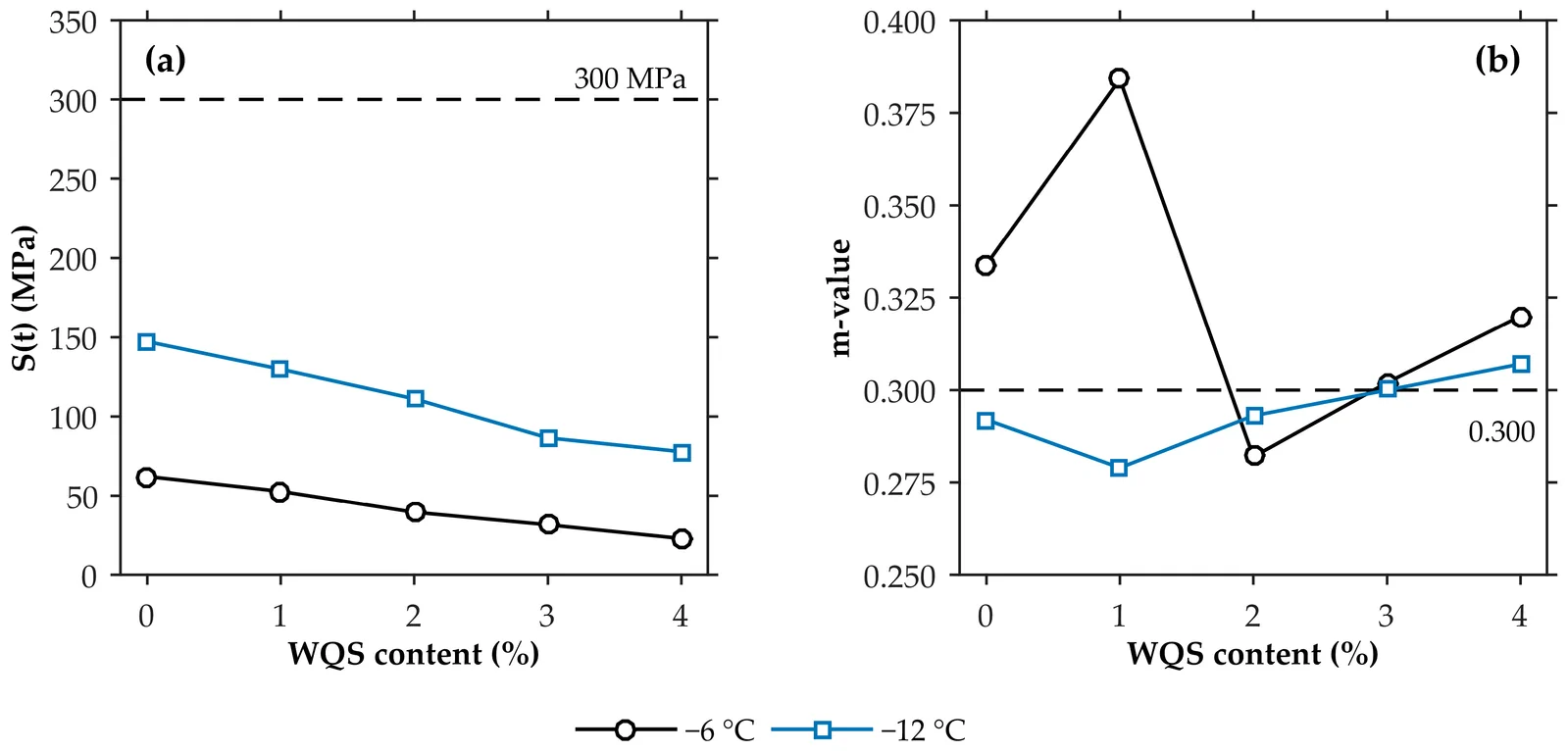
BBR test results: (**a**) creep stiffness and (**b**) m-Value of binders containing 0–4% WQS at −6 and −12 °C.

BBR tests conducted on the neat bitumen and WQS-modified binders showed that WQS incorporation affected the low-temperature rheological properties of the binders. The creep stiffness (S(t)) values of all binders remained below the Superpave specification limit of 300 MPa at both −6 °C and −12 °C, indicating that all binders satisfied the low-temperature stiffness requirement at both test temperatures (Figure 10a). Nevertheless, creep stiffness gradually decreased with increasing WQS content, with the lowest S(t) values obtained for the 4% WQS-modified binder. This reduction in creep stiffness suggests that the binders may be better able to relax thermally induced stresses at low temperatures, potentially reducing the risk of thermal cracking.

The m-value results indicated that the effect of WQS incorporation on stress relaxation was dependent on the modification level. At −6 °C, all binders except the 2% WQS-modified binder satisfied the Superpave requirement of m ≥ 0.300 (Figure 10b). The 1% WQS-modified binder exhibited a higher m-value than the neat bitumen, whereas the 3% and 4% WQS-modified binders showed slightly lower m-values than that of the neat bitumen while still satisfying the specification criterion. At the lower temperature of −12 °C, only the 3% and 4% WQS-modified binders satisfied the minimum m-value requirement. This finding indicates a more favorable stress relaxation response for binders with higher WQS contents under the lower test condition. The observed behavior may be associated with the fine particle size and high SiO_2_ content of WQS, which can influence the low-temperature rheological response of the bitumen matrix. At lower WQS contents, the incorporation of mineral particles may increase binder stiffness, which could contribute to the lower m-value observed for the 2% WQS-modified binder at −6 °C. At higher WQS contents, however, the combination of lower creep stiffness and compliance with the m-value criterion at −12 °C suggests a more favorable low-temperature response. Nevertheless, the specific microstructural mechanism responsible for this behavior cannot be determined solely from the BBR results.

The BBR results indicate that the influence of WQS incorporation on low-temperature performance depended on both the modification level and the performance criterion considered. While all binders satisfied the creep stiffness requirement at both test temperatures, compliance with the m-value criterion varied with WQS content and temperature. The 3% and 4% WQS-modified binders satisfied both criteria at −12 °C, whereas the 2% WQS-modified binder failed to meet the m-value requirement even at −6 °C. These findings highlight a trade-off between the 2% WQS content, which provided the most favorable high-temperature rheological performance in the DSR tests, and the 3–4% WQS contents, which exhibited more favorable low-temperature stress relaxation behavior. Therefore, both high- and low-temperature rheological properties should be considered when determining the appropriate WQS modification level.

### 3.6. Adhesion Tests

The Nicholson stripping and Vialit adhesion tests were conducted to evaluate the aggregate–binder adhesion performance of the neat and WQS-modified bitumen binders. The Nicholson stripping test results indicate that WQS incorporation did not cause a notable change in the stripping resistance of the binders. The bitumen coverage was approximately 90% for the neat bitumen and the binders containing 1%, 2%, and 4% WQS, whereas a slightly lower value of 85% was obtained for the 3% WQS-modified binder. Thus, the 3% WQS-modified binder exhibited a reduction of 5 percentage-point in the bitumen coverage compared with the neat bitumen, while the other WQS-modified binders showed no noticeable change. These results indicate that WQS incorporation did not adversely affect bitumen–aggregate adhesion, and that the overall resistance to water-induced stripping was generally maintained.

**Figure 11:**
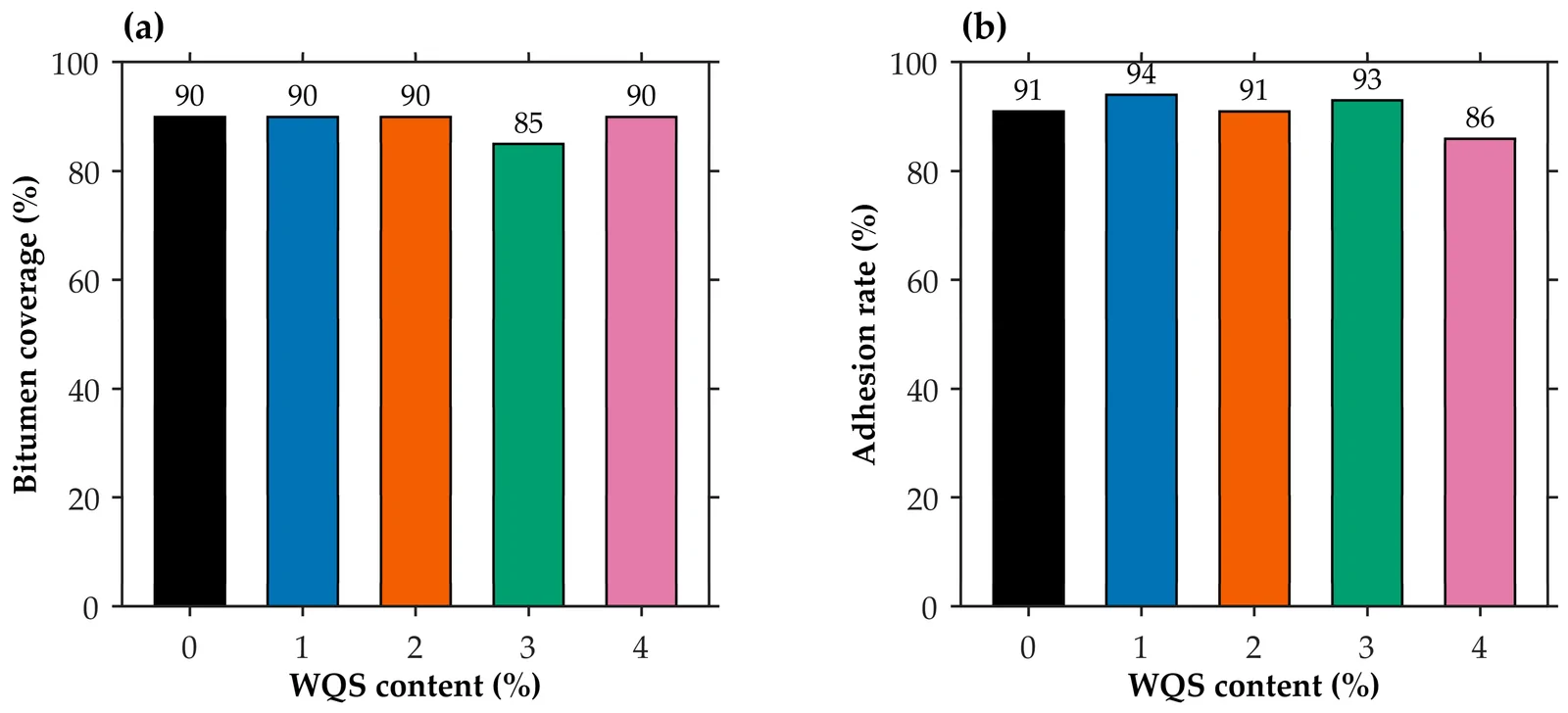
(**a**) Nicholson stripping test results. (**b**) Vialit adhesion test results.

The results obtained from the Vialit adhesion tests indicate that WQS incorporation improved the adhesion performance at certain modification levels. The highest adhesion rate was obtained for the 1% WQS-modified binder (94%), followed by the 3% WQS-modified binder (93%). The neat bitumen and the 2% WQS-modified binder exhibited similar adhesion rates of 91%. Compared with the neat bitumen, the adhesion rate therefore increased by approximately 3.3% at 1% WQS and 2.2% at 3% WQS, while no change was observed at 2% WQS. In contrast, the adhesion rate decreased to 86% for the 4% WQS-modified binder, corresponding to a reduction of approximately 5.5% relative to the neat bitumen and an aggregate loss of 14%, thereby exceeding the maximum aggregate loss criterion of 10% specified in the Turkish Highway Technical Specifications [77]. The measured aggregate loss was therefore 4 percentage points above the specified maximum limit. This result suggests that a high WQS content may adversely affect adhesion performance, potentially due to changes in the interaction between the binder and aggregate surfaces. These findings can be considered in relation to the chemical composition and particle morphology of WQS. The high SiO_2_ content (68.19%) identified by XRF confirms the silica-rich composition of WQS. In addition, the presence of CaO (4.59%) and Al_2_O_3_ (12.00%) may contribute to interactions at the bitumen–aggregate interface. Previous studies have reported that mineral phases containing CaO can contribute to stronger interactions between bitumen and aggregate and may improve adhesion under moisture-related conditions. Conversely, Li et al. [44] reported potential adhesion limitations for a quartz-rich iron-tailings filler with high SiO_2_ content, indicating that the mineral composition and filler content can significantly influence asphalt–filler interactions. Therefore, maintained or improved adhesion performance observed at WQS contents of 1–3% may be associated not only with the physical contribution of the mineral particles but also, potentially, with the contribution of mineral oxides to interfacial interactions. The Nicholson stripping and Vialit adhesion test results are presented in Figure 11a and Figure 11b, respectively.

The SEM images showed that WQS particles exhibited an irregular surface morphology and angular particle geometry. Such particle characteristics may promote physical interactions between the mineral particles and the bitumen matrix and may contribute to binder adhesion to the aggregate surface. However, the reduction in adhesion observed at 4% WQS indicates that a higher modifier content may adversely affect the binder–aggregate interfacial response. Since the present SEM analysis characterized the morphology of the WQS particles but did not directly examine their dispersion within the bitumen matrix, the underlying microstructural mechanism cannot be confirmed from the available data. Moreover, the present XRF and SEM analyses do not provide direct evidence of chemical bonding between WQS and bitumen; therefore, the proposed interfacial effects should be interpreted as possible mechanisms rather than confirmed chemical interactions.

The adhesion test results indicate that WQS contents of 1–3% maintained satisfactory adhesion performance, whereas the 4% WQS content resulted in a noticeable reduction in Vialit adhesion and failed to satisfy the specified aggregate loss criterion. In particular, the Vialit adhesion rate increased by up to 3.3% at the lower WQS contents, whereas a 5.5% reduction was observed at 4% WQS compared with the neat bitumen. This behavior is generally consistent with the DSR results, which identified 2% WQS as the most favorable modification level in terms of high-temperature rheological performance. The findings therefore suggest that the performance of WQS as a bitumen modifier depends on its mineral characteristics and modification level, while the possible role of particle dispersion requires further investigation. Accordingly, determining an appropriate WQS content is important for achieving a balance between high-temperature rheological performance and bitumen–aggregate adhesion.

### 3.7. Screening-Level Environmental and Economic Assessment

A screening-level environmental and economic assessment was conducted based on 1 tonne of total binder to evaluate the potential benefits of WQS incorporation. WQS was added at 0–4% by weight, with the corresponding reduction in bitumen content considered in the calculations. As WQS was obtained as an industrial waste material, its acquisition cost was assumed to be negligible. The WQS preparation process included natural solar drying and mechanical grinding. Assuming that drying was performed using solar energy, no direct energy consumption was assigned to the drying stage. Solar drying processes generally require substantially lower external energy input than conventional thermal drying approaches, although their effectiveness depends strongly on climatic and operating conditions [78]. Accordingly, only the grinding energy was considered in the present assessment. Based on a grinder power of 1.5 kW and a grinding time of 6.9 h/t WQS, the specific grinding energy was calculated as 10.35 kWh/t WQS [49]. The corresponding energy requirements for each WQS-modified binder are presented in Table 6.

The economic assessment was based on the current industrial electricity tariff and the 50/70 penetration-grade bitumen unit price in Türkiye. The electricity price was taken as 5.464 TL/kWh from the tariff published by the Energy Market Regulatory Authority (EPDK) [79], while the bitumen price was taken as 25,054 TL/t [80]. The total cost was calculated by considering the cost of the remaining bitumen and the electricity consumed during WQS grinding. For the environmental assessment, bitumen production was assigned an emission factor of 365 kg CO_2_-eq/t based on Gruber and Hofko (2023) [81]. Electricity-related emissions were calculated using an emission factor of 0.469 kg CO_2_-eq/kWh reported by the Republic of Türkiye Ministry of Energy and Natural Resources [82]. Total greenhouse gas (GHG) emissions were based on the emissions associated with bitumen production and WQS grinding.

Increasing the WQS content progressively reduced both the binder cost and GHG emissions. As expected from the reduction in virgin bitumen content, the 4% WQS-modified binder provided the greatest estimated economic and environmental savings, reducing the total binder cost by approximately 3.99% and GHG emissions by approximately 3.95% relative to the reference binder. These results indicate that WQS incorporation can provide economic and environmental benefits by reducing virgin bitumen consumption, while the additional energy demand associated with grinding remains relatively small. The detailed results of the screening-level economic and environmental assessment are presented in Table 6.

However, the economic and environmental benefits should be interpreted together with the experimental performance results. Although increasing WQS content continuously reduced the estimated binder cost and GHG emissions, higher WQS contents did not necessarily provide superior overall binder performance. Therefore, the selection of an appropriate WQS content requires a balance between material savings, environmental benefits, and engineering performance. It should also be noted that the present analysis is a simplified screening-level assessment rather than a full life-cycle assessment. Transportation, storage, mixing, construction, pavement maintenance, service life, and end-of-life stages were not considered. In practice, the energy demand associated with WQS drying may vary depending on climatic conditions and the availability of passive drying, and this variability was not explicitly modeled in the present screening-level assessment.

### 3.8. Relationship Between Economic–Environmental Benefits and Binder Performance

The economic and environmental results presented in Table 6 should be evaluated together with the physical and rheological properties of the WQS-modified binders. Increasing WQS content continuously reduced virgin bitumen consumption, GHG emissions, and binder cost; however, the overall binder performance depended on the balance among stiffness, ductility, aging behavior, and temperature-dependent rheological properties.

The decrease in penetration and the increase in softening point with increasing WQS content indicate a progressive stiffening of the binder and an improvement in high-temperature deformation resistance, consistent with the DSR results. For example, at 4% WQS, penetration decreased by approximately 42.7%, and the softening point increased by approximately 7.6% compared with the neat bitumen. Thus, the reduction in virgin bitumen consumption was accompanied by an improvement in high-temperature binder performance. However, this increase in stiffness was accompanied by a reduction in ductility, particularly at 3% and 4% WQS contents. The corresponding reductions in ductility were approximately 22.0% and 46.8%, respectively. This finding indicates that increasing WQS content beyond the favorable modification range may adversely affect binder deformability and potentially increase susceptibility of the binder to cracking.

From an environmental and economic perspective, increasing WQS content from 0% to 4% resulted in progressively greater reductions in estimated GHG emissions and binder cost. At 4% WQS, the estimated binder cost and GHG emissions decreased by approximately 3.99% and 3.95%, respectively, relative to the reference binder. Although 4% WQS provided the greatest direct economic and environmental benefit, its pronounced reduction in ductility and its comparatively lower rheological performance at high temperatures indicate that the highest investigated WQS content does not necessarily provide the most favorable overall binder performance. The aging and BBR results further demonstrate that the selection of an appropriate WQS content should consider both aging-related behavior and low-temperature performance, in addition to initial physical and high-temperature rheological properties.

The results indicate that intermediate WQS contents provide a more balanced combination of binder performance and sustainability benefits. In particular, 2% WQS appears to provide the most favorable overall balance, especially considering its high-temperature rheological performance, while WQS contents of 3–4% showed certain advantages under low-temperature conditions. Therefore, WQS represents a promising sustainable alternative for partial bitumen replacement, provided that its dosage is selected according to the specific performance requirements of the binder.

## 4. Conclusions

This study investigated the feasibility of using waste quartz sand (WQS), generated as a by-product of glass production, as a mineral-based modifier for bitumen through conventional binder characterization and Superpave performance testing. The main conclusions drawn from the experimental investigation and screening-level environmental and economic assessment are summarized as follows:

- WQS incorporation substantially influenced the physical and rheological properties of bitumen. The changes in penetration, softening point, together with the DSR, BBR, and adhesion test results, demonstrated that the effect of WQS was strongly dependent on the modification level. For example, at 2% WQS, penetration decreased by 37.4%, and the softening point increased by 4.2% relative to the neat bitumen, indicating a clear modification of binder consistency and high-temperature response.
- The 2% WQS content provided the most favorable overall high-temperature rheological performance. This dosage exhibited the most favorable combination of complex shear modulus, phase angle, and rutting parameter, particularly after RTFO conditioning. After short-term aging, the 2% WQS-modified binder satisfied the G*/sin(δ) criterion up to a maximum tested temperature of 82 °C, which was 12 °C higher than the maximum tested temperature at which the criterion was satisfied by the neat bitumen and the binders containing 1%, 3%, and 4% WQS. After RTFO+PAV conditioning, the lowest tested temperature at which the G*·sin(δ) criterion was satisfied was 4 °C for the 2% WQS-modified binder, compared with 16 °C for the neat bitumen. This result indicates a favorable fatigue-related response after long-term aging.
- Higher WQS contents showed advantages under specific low-temperature conditions. In the BBR tests, all binders satisfied the creep stiffness requirement at −6 °C and −12 °C, while the 3% and 4% WQS-modified binders were the only binders satisfying the m-value criterion at −12 °C. Thus, increasing the WQS content beyond 2% may provide benefits for low-temperature stress relaxation, although this did not translate into superior overall rheological performance.
- The highest WQS content did not provide the best overall performance. Although the 4% WQS-modified binder provided the greatest reduction in virgin bitumen consumption and consequently the highest estimated economic and environmental benefits, its ductility decreased by 46.8%, and its Vialit adhesion rate decreased by approximately 5.5% relative to the neat bitumen. These results indicate that maximizing WQS content is not necessarily desirable from an overall performance perspective.
- Based on the combined performance criteria, approximately 2% WQS can be considered the most favorable modification level among the investigated contents. This dosage provided the best balance between high-temperature rheological performance, aging-related behavior, low-temperature performance, adhesion, and sustainability considerations. At this modification level, the screening-level assessment also indicated reductions of approximately 2.00% in binder cost and 1.97% in GHG emissions. Nevertheless, the most favorable dosage may vary depending on the specific performance requirements and climatic conditions of the pavement application.
- The screening-level environmental and economic assessment demonstrated the potential sustainability benefits of WQS utilization. The progressive replacement of virgin bitumen with WQS reduced both estimated binder cost and GHG emissions, while the additional grinding energy remained relatively small. At 4% WQS, the estimated reductions reached 3.99% for binder cost and 3.95% for GHG emissions. These findings support the potential of WQS as a waste-derived material for more sustainable bitumen modification.

Overall, the findings demonstrate that WQS has potential as a sustainable mineral-based modifier for bitumen and can contribute to the valorization of glass-industry waste while reducing virgin bitumen consumption. The main contribution of this study is the integrated evaluation of WQS-modified binders in terms of conventional properties, temperature-dependent rheological behavior, aging response, low-temperature performance, aggregate–binder adhesion, and screening-level environmental and economic benefits. The results further demonstrate that the highest waste content does not necessarily provide the best engineering performance and that an intermediate WQS content, particularly around 2%, can provide a more favorable overall balance.

### Limitations and Future Work

Although the test results demonstrate the potential of WQS as a bitumen modifier, several limitations should be considered when interpreting the findings. The investigation was limited to B50/70 bitumen, WQS particles finer than 0.075 mm, and WQS contents of 0–4% by weight. Further studies using different penetration grades, particle-size distributions, and wider modification ranges would help clarify the applicability and dosage sensitivity of WQS under different binder conditions.

The experimental program provided comprehensive information on the physical, rheological, aging, and adhesion characteristics of the modified binders. The DSR evaluation was conducted at discrete temperature intervals of 6 °C for the unaged and short-term-aged binders and 3 °C for the long-term-aged binders. Therefore, the reported temperature-based comparisons represent directly measured test points rather than interpolated continuous critical temperatures. Future studies using smaller temperature increments could provide a higher-resolution determination of transition or critical temperatures. In addition, advanced rheological analyses such as multiple stress creep recovery (MSCR) and linear amplitude sweep (LAS) could not be performed due to equipment limitations. Future studies incorporating MSCR could provide additional insight into the non-linear high-temperature response and recovery characteristics of WQS-modified binders, while LAS testing could be used to quantitatively evaluate fatigue resistance and damage development. In addition, Cole–Cole and Black Space analyses could provide further information on the thermo-rheological behavior and structural changes in the modified binders.

Another limitation is that the interaction mechanisms between WQS and the bitumen matrix were not directly investigated at the microstructural or molecular level. Future research using techniques such as Fourier transform infrared spectroscopy (FTIR), X-ray diffraction (XRD), atomic force microscopy (AFM), or fluorescence microscopy could help clarify the physical and possible chemical interactions between WQS and bitumen and directly assess particle dispersion and possible agglomeration within the binder matrix. Surface free energy (SFE) analysis and Wilhelmy plate measurements would also be valuable in future studies to quantitatively evaluate the effects of WQS on surface energetics, wettability, and moisture-related binder–aggregate adhesion.

Although WQS has previously been investigated at the asphalt-mixture level, the favorable binder-modification level identified in the present study, particularly around 2% WQS, requires further mixture-level validation using the same modification approach before practical implementation.

Finally, although RTFO and PAV procedures were used to simulate short- and long-term aging, the long-term performance of WQS-modified binders under actual field conditions was not investigated. Field trials and long-term monitoring under different climatic conditions would therefore be valuable for validating the laboratory findings and assessing the practical durability of WQS-modified binders.

## Figures and Tables

**Table 1:** Basic properties of base bitumen.

Property	Test Result
Penetration (0.1 mm)	59.65
Specific gravity	1.033
Softening point (°C)	55.70
Flash point (°C)	295

**Table 2:** Chemical composition of WQS determined by XRF analysis.

Chemical Composition	(%)	Chemical Composition	(%)
SiO_2_	68.194	BaO	0.03
Al_2_O_3_	12.004	Cl	0.026
K_2_O	5.989	MnO	0.014
CaO	4.585	Cr_2_O_3_	0.013
MgO	1.673	SrO	0.01
Fe_2_O_3_	0.733	Rb_2_O	0.007
TiO_2_	0.623	ZnO	0.005
P_2_O_5_	0.185	PbO	0.004
F	0.161	NiO	0.003
Na_2_O	0.088	Y_2_O_3_	0.003
ZrO_2_	0.075	Nb_2_O_5_	0.001
SO_3_	0.053	L.O.I ^1^	5.521

^1^ L.O.I: Loss on Ignition.

**Table 3:** Results of conventional binder tests.

WQS (%)	Specific Gravity	Penetration (0.1 mm)	Softening Point (°C)	Flash Point (°C)	Ductility (cm)
0	1.033	59.65	55.70	295	141
1	1.035	49.20	55.55	295	141
2	1.058	37.35	58.05	280	141
3	1.059	34.60	58.90	280	110
4	1.060	34.15	59.95	285	75

**Table 4:** Results of storage stability tests.

WQS (%)	Softening Point (°C)				Penetration (0.1 mm)			
	Before Storage	SP_top_	SP_bottom_	ΔSP	Before Storage	P_top_	P_bottom_	ΔP
0	55.70	56.9	57.0	0.1	59.65	44.3	44.1	0.2
1	55.55	57.6	57.5	0.1	49.20	42.9	43.9	1.0
2	58.05	59.3	59.8	0.5	37.35	40.5	42.0	1.5
3	58.90	62.3	62.6	0.3	34.60	49.8	50.3	0.5
4	59.95	63.3	63.9	0.6	34.15	56.1	59.4	3.3

**Table 5:** Results of RTFO tests.

WQS (%)	RTFO Mass Change (%)	Softening Point After RTFO (°C)	Softening Point Difference (°C)	Penetration After RTFO (0.1 mm)	Penetration Ratio (%)
0	−0.028	52.95	−2.75	65.30	109.47
1	−0.03	61.45	5.90	29.20	59.35
2	−0.02	66.75	8.70	20.00	53.55
3	−0.04	72.75	13.85	23.40	67.63
4	−0.03	75.55	15.60	27.40	80.23

**Table 6:** Screening-level economic and environmental assessment of WQS-modified bitumen.

Binder Type	0-WQS	1-WQS	2-WQS	3-WQS	4-WQS
WQS (kg/t)	0	10	20	30	40
Bitumen (kg/t)	1000	990	980	970	960
Grinding energy (kWh/t)	0	0.104	0.207	0.311	0.414
Bitumen cost (TL/t)	25,054.00	24,803.46	24,552.92	24,302.38	24,051.84
Grinding cost (TL/t)	0	0.568	1.131	1.699	2.262
Total cost (TL/t)	25,054.00	24,804.03	24,554.05	24,304.08	24,054.10
Cost reduction (%)	–	1.00	2.00	2.99	3.99
Bitumen GHG (kg CO_2_-eq/t)	365	361.350	357.700	354.050	350.400
Grinding GHG (kg CO_2_-eq/t)	0	0.049	0.097	0.146	0.194
Total GHG (kg CO_2_-eq/t)	365	361.399	357.797	354.196	350.594
GHG reduction (%)	–	0.99	1.97	2.96	3.95

## Data Availability

The data presented in this study are included in the article. Further inquiries can be directed to the corresponding author.

## Abbreviations

The following abbreviations are used in this manuscript:

WQS	Waste Quartz Sand
DSR	Dynamic Shear Rheometer
BBR	Bending Beam Rheometer
PAV	Pressure Aging Vessel
RTFO	Rolling Thin-Film Oven
SEM	Scanning Electron Microscopy
XRF	X-Ray Fluorescence
GHG	Greenhouse Gas
SBS	Styrene-Butadiene-Styrene
SBR	Styrene-Butadiene-Rubber
EVA	Ethylene-Vinyl-Acetate
PE	Polyethylene
PP	Polypropylene
TS	Turkish Standard
EN	European Norm
ISO	International Organization for Standardization
MEITAM	Advanced Technology Education, Research and Application Center
AFM	Atomic Force Microscopy
FTIR	Fourier Transform Infrared Spectroscopy
XRD	X-Ray Diffraction
MSCR	Multiple Stress Creep Recovery
LAS	Linear Amplitude Sweep
SFE	Surface Free Energy
